# Translation and cross-cultural adaptation of the Work Rehabilitation Questionnaire (WORQ) into Danish

**DOI:** 10.3389/fresc.2023.1134039

**Published:** 2023-06-08

**Authors:** Katrine Baltzer Thygesen, Mette Korshøj, Ida Skovgaard Verpe, Lise Vestergaard, Reuben Escorpizo, Ole Steen Mortensen

**Affiliations:** ^1^ Department of Occupational and Social Medicine, Holbæk Hospital, Part of Copenhagen University Hospital, Holbæk, Denmark; ^2^ Department of Rehabilitation and Movement Science, College of Nursing and Health Sciences, University of Vermont, Burlington, VT, United States; ^3^ Department of Public Health, Section of Social Medicine, University of Copenhagen, Copenhagen, Denmark

**Keywords:** WORQ, psychometrics, work, comorbidity, functioning ability, vocational rehabilitation (VR), ICF (international classification of functioning, disability, and health)

## Abstract

**Purpose:**

This study aimed to translate and cross-culturally adapt the work rehabilitation questionnaire (WORQ) into Danish to examine the internal consistency and test-retest reliability of the Danish WORQ and, second, to test the feasibility of WORQ in the Danish context of vocational rehabilitation.

**Methods:**

The translation was performed in a dual-panel approach. The panel consisted of a bilingual physician, a university student in psychology, a layperson, a specialist in social work and rehabilitation, and a professor in social medicine. The international classification of functioning, disability, and health (ICF) codes were cross-evaluated to secure that there was a high level of agreement of ICF codes for each specific WORQ item in the Danish and English version. The content validity was evaluated by the clinical physicians at an outpatient clinic in social medicine and by the case managers at a municipality job center. Data for the examination of the internal consistency and test-retest reliability were collected at the Holbæk municipality from citizens in the working age. The test-retest took place 14 days apart. The internal consistency and test-retest reliability were tested conducting Cronbach's alpha, intraclass correlation, and Spearman’s correlation analyses.

**Results:**

The panel experienced only minor challenges in the translation process, leading to minor modifications. The cross-evaluation of coding in the Danish WORQ compared with the initial English version only found small deviations, while the remaining coding agreed between the initial English and the Danish items. The panel argued to add sub-codes to culturally adapt to the transportation forms generally used in Denmark. The general perception among the participating patients and citizens at the job center as well as the clinical physicians and case managers was that the WORQ was easy, understandable, and meaningful.

**Conclusions:**

This study showed that the Danish WORQ have a high content validity and usability. Nonetheless, the Danish WORQ needs to be validated against well-acknowledged tools for assessing functional ability specific to work and in general.

## Introduction

In Denmark, as well as across the Western world, the demography of the workforce changes toward higher ages (1). This, in combination with the changes of paradigms regarding retirement, early retirement, and flex-job (employment on special conditions for employees with permanent reduced work ability) pointing toward the need of a higher employment rate and thus higher retirement ages are introduced (2, 3). In addition, these changes increase the need for the strategies for disability management to ensure the highest possible employment rate. One of the elements in the strategy for disability management is vocational rehabilitation, supporting all individuals across the working age to achieve the best possible work participation (4). Vocational rehabilitation aims to improve work ability, prevent chronicity of health-related impairments, decrease sick leave, and support the individual societal participation (5). The key element in the planning of vocational rehabilitation is an assessment of work demands as well as the workers resources. Previously, the balance between these elements has been described as the work ability; however, in the context of vocational rehabilitation, the resources may be defined as abilities of function and professional skills and values (6). In Denmark, vocational rehabilitation is handled by the case managers in the municipality job centers, where the focus is on returning to the labor market as soon as possible and to the greatest extent possible. Advice regarding heath issues in complex cases is given by the regional Department of Social Medicine. Thus, vocational rehabilitations are targeting the highest possible level of reestablishment of work functioning/work ability, and therefore are a comprehensive understanding of the multifaceted relations of the individual’s work and health resources needed (7).

The international classification of functioning, disability, and health (ICF), developed by the World Health Organization (8, 9), has previously been shown to provide an appropriate framework to capture the multifaceted relations of individual work and health resources (10, 11). The ICF provides a model for description and mapping of functionality. The ICF core sets are developed to operationalize the ICF codes that are specific to the context or predefined target group (12). To make use of the ICF core sets in a vocational rehabilitation context, the work rehabilitation questionnaire (WORQ) was developed (12). The WORQ is based on the biopsychosocial approach, and the two versions, interviewer and patient-administered, have shown to be feasible and provided high patient satisfaction (13, 14). The items in WORQ (www.myworq.org) cover specific ICF codes with relevance for work ability, and may therefore be used in the assessment and understanding of the need for vocational rehabilitation, target and fit of the initiatives for improvement of the functionality. Further studies are needed to clarify if WORQ has the potential to measure and evaluate the effect of the rehabilitation initiative.

In Denmark, there is a need for a common rehabilitation language and standardization of data on work resources to be used in the interdisciplinary vocational rehabilitation work, and therefore there is a need for a method for uniform assessment of work resources, such as WORQ. Although the WORQ is translated and culturally adapted to Dutch, German, and French (15, 16), the applicability to the Danish population and culture is lacking as the majority of the Danish population speaks Danish and the vocational rehabilitation context is unique for Denmark. This study aimed to translate and cross-culturally adapt the WORQ into Danish, and to examine the internal consistency and test-retest reliability of the Danish WORQ. Second, this study aimed to test the feasibility of the WORQ in the Danish context of vocational rehabilitation.

## Methods

### Design

The study was designed in two parts: initially: the translation, cross-cultural adaptation, and content validity testing; and second: the internal consistency, test-retest reliability, and construct validity of the WORQ in Danish. The process of translation and cross-cultural adaptation, the testing of content validity, and the examination of the internal consistency and test-retest reliability of the Danish WORQ will be reported in this paper, while the construct validity will be reported in another paper (17).

The WORQ is divided in two parts: the first part collects sociodemographic information, information on previous employments including work tasks, present rehabilitation plans including (i) need for retraining into a different occupational group; (ii) ongoing treatments; and (iii) sufficient support from family, present employer, and job center. The second part collects information on social, mental, and physical functioning from 40 items, with response categories ranging from 0 to 10, where 0 corresponds to no problem and 10 to the highest degree of being problematic. In addition, two items collect information on the total time used per week on getting ready in the morning, i.e., time spent in hours and minutes from getting out of bed until you leave your home and spent on participation in ongoing treatments, e.g., performance of specific training programs.

### Translation and cross-cultural adaptation

The translation was performed by a dual-panel approach (18). This approach has previously shown to not affect the psychometrics in comparison with the forward-backward method (19), and was shown to be the preferred method by laypersons (non-patient) and patients, which may positively affect the response rates and quality of the collected data. Although Danish and English cultures may be considered quite similar and thus it may not be difficult to reach conceptual equivalence, the contextual adaptation and user-acceptability are still crucial for ensuring the quality of the collected data.

**Figure 1:**
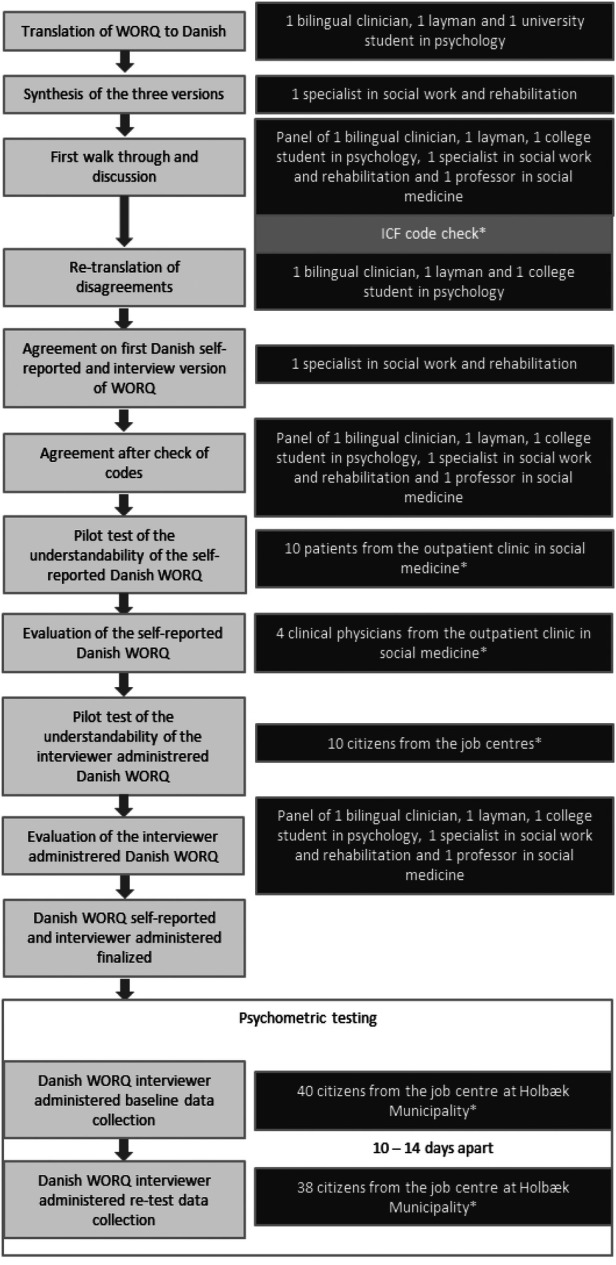
Overview of the translation to Danish and cross-cultural adaptation process. *Additional to the dual-panel approach.

The panel consisted of a bilingual physician, a university student in psychology, a layperson with excellent skills in Danish and English, a specialist in social work and rehabilitation, and a professor in social medicine. All members in the panel were Danish speaking and were familiar with the Danish vocational rehabilitation system. The dual-panel approach followed the procedure visualized in Figure 1, in brief translation by individuals, synthesis and discussion of translations, revision of translations where needed, agreement upon translation, followed by the cross-evaluation of coding, understanding and feasibility testing of patient- and interviewer-administered versions, revision of the translation and cultural adaptation where needed, and final agreement upon translation.

### Cross-evaluation of coding

The Danish WORQ was cross-evaluated against the original ICF coding for the initial version of the WORQ in English to avoid misunderstanding and translational errors. The cross-evaluation was performed by an evaluation of the level of agreement of ICF codes for each specific WORQ item in the Danish and English version. The codes for the initial version of the WORQ were provided by the head of the group developing the initial WORQ, Dr. Reuben Escorpizio.

### Content validity

Content validity was defined as the “relationship between a test’s content and the construct it is intended to measure” (20). The content validity for the patient-administered WORQ was evaluated by four clinical physicians and six of 10 invited patients at the outpatient clinic in social medicine. Also, the content validity for the interviewer-administered WORQ was evaluated by two case managers interviewing 10 citizens at the job center. The content validity was evaluated by the case managers at the job center, the patients, and the clinical physicians. They were asked to reflect upon whether the WORQ captured all facets of the work resources relevant for vocational rehabilitation by answering the question “*Is there any questions you would like to include? If yes, please state which.*”

### Feasibility of the self-administered questionnaire

The feasibility of the self-administered WORQ was tested by the patients summoned for an examination in the outpatient clinic in social medicine. The enrolled group of patients were sought to represent a variety of age, sex, educational level, and diagnosis. It was not possible to select on occupation, since all patients were without job at the time for the examination in the outpatient clinic. The patients were selected by the aforementioned criteria and were informed about the usability testing by an information sheet mailed jointly with the call letter for the examination at the outpatient clinic in social medicine. The patients were asked to fill in the pilot version of the self-administered WORQ in Danish and were thereafter asked to answer the usability questions: “*Did you experience any difficulty in understanding these questions?*” and “*Did the response categories make sense for you?*” to collect information on their perception of the questionnaires understandability, clarity, feasibility, and appropriateness.

### Feasibility of the interviewer-administered questionnaire

The feasibility testing of the interviewer-administered questionnaire was performed at the job center and in the outpatient clinic in social medicine. At the job center, the first author (KBT) performed the recruitment and initial information of the citizens. The enrolled citizens were interviewed by KBT and supervised by the case manager, using the interviewer-administered WORQ. After filling in the second version of the self-administered WORQ, the case managers at the job center were asked to answer the usability questions. Also, the clinical physicians were asked to complete an evaluation of the usability of the information provided by the self-administered WORQ after the examination of the patient filling in the questionnaire. The usability was evaluated by the following questions: “*When you have completed an examination and consultation of the patient, does the patient's answers in WORQ provide information you didn’t get otherwise?*”; “*Is there any questions in WORQ that does not make sense or you don’t understand? If yes, please state which items,*” *and “Is there any of the patientś answers that is a surprise to you or makes you wonder? If yes, please state which.”*

Six of 10 invited citizens at the job center were interviewed with the same questions as the patients in the outpatient clinic, based upon the second version of the interviewer-administered WORQ in Danish, the second version being the pilot version revised based on the usability testing at the outpatient clinic in social medicine. The case managers evaluated the usability of the interviewer-administered WORQ by answering the same questions as the clinical physicians were asked to answer.

### Test-retest reliability and internal consistency

The validation of the Danish WORQ took place at the Holbæk municipality. The case managers at the job center recruited working age clients, who were required to be competent in Danish to complete the Danish WORQ. The recruited citizens were required to make two appearances at the job center for completing the interviewer-administered version of WORQ administered by their case managers. A gap of 14 days between test-retest was chosen under the assumption that citizens would have forgotten their initial answers while at the same time alterations in functioning would be unlikely to have occurred.

In order to examine the internal consistency and test-retest reliability, the 40 WORQ items on functioning were divided into subscales that have earlier been derived in the translation, cross-cultural adaptation, and psychometric evaluation of the WORQ into Dutch (16). They derived the following seven subscales: cognition, physical, mood, activities of daily living (ADL), sensory, emotions, and social.

### Statistical analysis

No statistical analyses were performed for the translation and cross-cultural adaptation; however, the percentages of patients, clinical physicians, or case managers’ satisfaction and rating of usability are reported.

With regard to the validation, descriptive statistics were performed on the background—and sociodemographic variables. Normality of the data was examined through histogram analysis and the Kolmogorov–Smirnov test (20).

The parametric data were correlated by Pearson's correlation and non-parametric data by Spearman's correlation (21).

To make sure the Dutch subscales matched the Danish translation and cross-cultural adaptation, a correlation matrix of the items in each subscale was made.

The internal consistency of the Danish WORQ was examined with a Cronbach's alpha analysis on the subscale-divided data.

The test-retest reliability on items were examined by first conducting Spearman's correlation on the initial test-retest answers on every single item of the 40 WORQ items on functioning.

Second, intraclass correlation (ICC) on scales was performed on the initial test-retest answers divided into the seven subscales.

Lastly, ICC was performed on the total WORQ score for test-retest, that is, not divided into the subscales.

All statistical analyses were performed with the software package IBM SPSS Statistics for Mac, version 26.0. The statistical level of significance was set at an alpha level of 0.05.

This study was approved by the ethical committee of Region Sjaelland, Denmark, and the Danish Data Protection Agency (journal no. REG-073-2018 file no.: 19-000079/2018-073). Prior to voluntary participation, the participants signed an informed consent in accordance to the Declaration of Helsinki.

## Results

### Translation and cross-cultural adaptation

The panel experienced only minor challenges in the translation process, leading to the discussion of the response categories and modification in part 1 of the following: item 7 (highest level of education) was adapted to fit the Danish educational system; item 6 (current vocational rehabilitation activities) and 14 (“what kind of work or vocational intervention are you receiving now?”) was adapted to fit the activities offered in the Danish vocational rehabilitation program; item 4 “not applicable” was not directly translated into “ikke anvendelig” but to the more meaningful “aldrig arbejdet.” In part 2, in items 1–38, the “complete problem” was not directly translated to “komplet problem” but to the more accurate “i højeste grad et problem.”

### Cross-evaluation of coding

The cross-evaluation of coding in the Danish WORQ compared with the initial English version only found small deviations leading to the following changes: (i) item 6 of the Danish version did not hold all the relevant codes, and the missing codes were added without changing the wording of the item; (ii) item 17 was miscoded; this was corrected and the item re-worded to fit the coding. All other coding had a 100% match between the initial English and the Danish items.

Due to the cultural adaptation into a Danish context, where it was thought essential to collect additional information of the ability to use different transportation modes, the panel argued to add sub-codes to item 33 (ICF code d470) and 34 (ICF code d475) with the corresponding additional items asking to state the ability to the following: (i) *Private transport by a relative driving the car* (sub-code d4701), (ii) *Public transport by use of bus, train, or such* (sub-code d4702), (iii) *Drive a car, scooter, moped, motorbike* (d4751), and (iv) *Drive a bike* (d4750).

### Content validity

The clinical physicians evaluating the content validity highlighted the lack of questions regarding (i) non-motoric difficulties such as stress or bowel problems, (ii) use of tobacco, alcohol, and drugs, (iii) self-rated workability, (iv) the patients’ expectations for the examination in the outpatient clinic in social medicine, and (v) more specific information on diet. None of these suggestions for additional questions led to any added items in the questionnaire. However, the information is given to the researcher group behind WORQ, and the suggested items are now included as an addendum to WORQ in the Department of Social Medicine at Holbæk Hospital.

The evaluation of the content validity highlighted the lack of questions regarding (i) whether the citizen holds a driver’s license or not, (ii) non-educationally based working skills, such as autodidactically trained, and (iii) abuse of drugs or abusive behavior. Also, the response category of never married did not cover those co-habituating during longer periods of life, but not presently. Although not specifically mentioned in the wording of question 36, abusive behaviors are reflected in the ICF codes linked to question 36 in WORQ, and due to the wording in the initially developed WORQ, it was chosen to not be changed in the Danish version. The case managers were also asked to evaluate the interviewer-administered WORQ, and they responded positively as the perspectives of using WORQ provided additional relevant information to support their evaluation of initiatives targeted functionality in relation to work participation.

### Usability of the self-administered questionnaire

Of the recruited 10 patients, 6 showed up at the outpatient clinic in social medicine. They covered a variety of age, sex, and educational level, (Table 1).

**Table 1:** Descriptive information of the enrolled participants in the usability testing of the self-administered questionnaire, *n* = 6.

	Mean	SD	*n* (%)
Age (years)	48.1	9.6	
Sex (% females)			4 (67%)
Educational level (% medium level or above)			3 (50%)

The general perception among the participating patients and citizens at the job center was that the WORQ was easy and understandable and they all found the response categories meaningful.

The patients stated that they had trouble in understanding 11 of the 42 items, corresponding to 26.2%, and 3 of the 42 (7.1%) response categories did not made sense for the participating patients.

Thus, the following modifications and additional cultural adaptations were applied to the response categories: item 5 was rephrased to clarify the meaning of the response category regarding un-employment due to participation in vocational rehabilitation; item 6 was culturally adapted as patients being on sick leave are not allowed to apply for new jobs while being on sick leave (22); item 9 was rephrased and limited to only include the branch and not the company and industry; in item 10, the past tense of present work tasks was added; item 14 was specified by adding examples of initiatives for return to work; and item 17 was adapted culturally by supplementing examples of support functions.

### Usability of the interviewer-administered questionnaire

The clinical physicians found the WORQ easy to understand in general. However, the clinical physicians stated that they had trouble in understanding 2 of the 42 items, corresponding to 4.8%. Also two (4.8%) of the response categories were not meaningful for the clinical physicians. However, no adaptations were applied based on the feedback from the clinical physicians. Although the clinical physicians did not think that the information collected by the WORQ provided any information that was not provided otherwise, they still stated that some of the answers given by the patients surprised them or gave rise to wondering. The broad scope of WORQ shed light on symptoms from other organ systems than those that were mentioned as reason for sick leave, and also additional information on social relations.

Based on the evaluation of the usability testing of the interviewer-administered WORQ at the job center, only smaller linguistic adaptations were made for clarifying question 4, 5, 8, and 18.

### Test-retest reliability and internal consistency

A total of 68 clients were recruited. Of the 68 recruited, 28 failed to appear leaving us with 40 participants. Of the 40 participants performing the initial test, 38 showed for the retest. The characteristics of the population are shown in Table 2. There was an approximately equal distribution between males and females, and the mean age of participants was 42 years. None of the participants were working either due to health, ongoing vocational rehabilitation, or due to other reasons. Everyone had finished primary school, but no one had a university/college degree or higher levels of education. A total of 31 out of the 40 (72.5%) participants were engaging in programs related to preparation for employment such as apprenticeship or internship.

**Table 2:** Characterization of the population.

	Total population *n* = 40			
	Mean	SD	Range	*n* (%)
Age	42.40	7.87	28–60	
Sex				
Male				19 (47.5)
Female				21 (52.5)
Occupational status				
Full time employed				0 (0)
Part time employed				0 (0)
On modified or light duty				0 (0)
Not working due to health	* *	* *	* *	14 (35)
Not working due to ongoing vocational rehabilitation				8 (20)
Not working due to other reasons				18 (45)
Educational level				
Less than primary school				0
Primary school				13 (32.5)
High school/secondary school				8 (20)
Vocational education				13 (32.5)
College				6 (15)
University				0 (0)
Post-graduate degree				0 (0)
Vocational rehabilitation program				
Engaging in programs related to preparation for employment such as apprenticeship or internship				29 (72.5)
Engaging in vocational training activities such as in acquiring knowledge and skills for a job, including school training				0 (0)
Engaging in activities to secure or maintain current job				2 (5)
Looking for a (new) job or work				0 (0)
None of the abovementioned				9 (22.5)

Spearman's correlation showed good to excellent correlations on almost all of the 40 WORQ items with coefficients ranging from 0.391 to 0.855), all significant at the 0.05 level. Only 3 correlations were non-significant, ranging from 0.279–0.317 (Table 3).

**Table 3:** Test-retest correlation analysis, *n* = 38.

WORQ item	Correlation coefficient (*p*-value)	WORQ item	Correlation coefficient (*p*-value)
WT1	0.702 (0.000)	WT25	0.446 (0.005)
WT2	0.527 (0.001)	WT26	0.551 (0.000)
WT3	0.617 (0.000)	WT27	0.620 (0.000)
WT4	0.653 (0.000)	WT28	0.683 (0.000)
WT5	0.406 (0.011)	WT29	0.395 (0.014)
WT6	0.734 (0.000)	WT30	0.445 (0.005)
WT7	0.842 (0.000)	WT31	0.609 (0.000)
WT8	0.428 (0.007)	WT32	0.422 (0.013)
WT9	0.682 (0.000)	WT33	0.411 (0.010)
WT10	0.726 (0.000)	WT33a	0.413 (0.010)
WT11	0.651 (0.000)	WT33b	0.685 (0.000)
WT12	0.660 (0.000)	WT34	0.628 (0.000)
WT13	0.599 (0.000)	WT34a	0.460 (0.008)
WT14	0.666 (0.000)	WT34b	0.801 (0.000)
WT15	0.640 (0.000)	WT35	0.471 (0.003)
WT16	0.718 (0.000	WT36	0.425 (0.008)
WT17	0.279 (0.09)	WT37	0.556 (0.000)
WT18	0.670 (0.000)	WT38	0.855 (0.000)
WT19	0.766 (0.000)	WT39	0.307 (0.061)
WT20	0.692 (0.000)	WT40	0.565 (0.000)
WT21	0.633 (0.000)	WT41a	0.612 (0.000)
WT22	0.684 (0.000)	WT41b	0.391 (0.024)
WT23	0.450 (0.005)	WT42	0.539 (0.001)
WT24	0.509 (0.001)	WT42b	0.317 (0.100)

WORQ, work rehabilitation questionnaire.

Significant results are marked with italics.

The internal consistency assessed by the Cronbach's alpha analysis showed satisfying results with most of the coefficients for the seven subscales in the area of 0.7–0.9. Only the subscale regarding sensory did not show good internal consistency with a coefficient of 0.6. See Table 4. For the total sum score of the 40 WORQ items, the Cronbach's alpha coefficient was 0.94.

**Table 4:** Reliability: internal consistency, *N* = 40.

Subscales (no. of items)	Internal consistency (Cronbach's alpha)	Test-retest reliability, ICC (95% CI)
Factor 1: Cognition (9)	0.87	0.93 (0.89–0.96)
Factor 2: Physical (8)	0.84	0.90 (0.84–0.95)
Factor 3: Mood (3)	0.76	0.83 (0.72–0.91)
Factor 4: ADL (5/7 with a/b)	0.74	0.82 (0.70–0.90)
Factor 5: Sensory (5)	0.60	0.78 (0.66–0.87)
Factor 6: Emotions (3)	0.73	0.85 (0.76–0.91)
Factor 7: Social (7/9 with a/b)	0.80	0.86 (0.78–0.92)
Total sum score of 40 items	0.94	0.91 (0.83–0.95)

ICC, intraclass correlation.

The test-retest reliability assessed by ICC also yielded great results on all seven subscales, and the total sum score of the 40 WORQ items with coefficients ranging from 0.78 to 0.96. See Table 4.

## Discussion

This study performed a translation and cross-cultural adaptation of the WORQ into Danish. As previously recommended (23, 24), a dual-panel approach was successfully applied for the translation and cross-cultural adaptation of WORQ from English to Danish. Moreover, the results from the feasibility testing of the Danish WORQ showed that the Danish WORQ was perceived as an easy and understandable tool for assessing self-reported functioning in relation to work by the participating patients and clinical physicians at a Danish outpatient clinic in social medicine, as well as the citizens and case managers at a job center. Hence, the Danish WORQ is considered ready to validate and conduct feasibility test in Danish contexts.

By the use of the biopsychosocial framework, the WORQ functions as a tool for describing the workability and general functioning among individuals (7, 12, 14). Therefore, WORQ may also function as a tool for the evaluation of vocational rehabilitation initiatives targeted functionality in relation to work participation. However, in order to reach the best potential for use of the assessed functionality by WORQ, proper linguistic translation and appropriate cultural adaptation are essential to achieve individuals to comply and give context-specific information. Yet, there is a need for the translation and cross-cultural adaptation to be applied to the initial WORQ and ICF coding to maintain the comparability across countries (25). In the translation and cross-cultural adaptation of the WORQ into Danish, the ICF codes were cross-evaluated against the English WORQ with a 100% match; however, the Danish WORQ holds additional codes as sub-codes were added to items 33 and 34 to appropriately reflect relevant modes of transportation in a Danish context. Furthermore, did the usability testing performed for the self-administered questionnaire led to cultural adaptations of the response categories to fit to the Danish context of legislation regarding job application while on sick leave and by giving specific and recognizable examples of initiatives for return to work and support functions.

Although the WORQ holds a variety of items and might seem long, the multifaceted assessment of functioning enables overviews of complex and joint problems owed to co-morbidities. The information collected by WORQ are therefore relevant for supporting the planning of initiatives and strategies for work participation, such as vocational rehabilitation, where a comprehensive understanding of the multiple relations of individual work and health resources are needed (26). These findings are in accordance with similar studies in Turkey and Switzerland (27, 28).

### Methodological considerations

It is a strength that the usability of the Danish WORQ was tested for both the self-administered and interviewer-administered versions; it is also considered a strength that the usability testing was performed by two different settings (an outpatient clinic in social medicine and a job center) involved in the initiatives targeted functionality in relation to work participation. However, this study also holds some limitations, such as that the usability testing was not performed among occupational therapists or physiotherapists who may be involved in initiatives targeted functionality in relation to work participation, such as vocational rehabilitation. Also, the usability testing was not performed among non-patients or citizens who are not enrolled at the job center; thus, the usability testing was not performed among a population of employed workers without any functional limitations.

### Practical implications

The WORQ provides an easily understandable tool for collecting information on work and general functioning by the use of the biopsychosocial framework, and thus including physical, mental, and social functionality. Thus, the WORQ offers a multifaceted assessment of functioning, enabling overview of complex and joint functionality limitations. On this basis, the WORQ may make a common understanding and terminology available for all stakeholders involved in initiatives targeted functionality in relation to work participation. In addition, the WORQ also holds potential as a screening and/or evaluation tool for support and targeting of initiatives targeted functionality in relation to work participation.

## Conclusion

The Danish WORQ was found to be reliable and to have a high content validity and usability among patients and clinical physicians in an outpatient clinic in social medicine. Nonetheless, the Danish WORQ needs to be validated against well-acknowledged tools for assessing functionality specific to work and in general.

## Data Availability

The raw data supporting the conclusions of this article will be made available by the authors, without undue reservation.
